# 

**Published:** 1871-07

**Authors:** 


### NOTICE.

Reviews of Books, and some Original Articles designed for this number, have been crowded out for want of space. These will appear in the next number.

### Books and Pamphlets Received.

A practical treatise on the diseases of infancy and childhood. By Thomas Hawkes Tanner, M. D., F. L. S. Third American edition. From the last London edition, revised and enlarged by Alfred Meadows, M. D., Lond· Philadelphia: Lindsay & Blakiston, 1871. Buffalo: Theo. Butler & Son.

Opium and the Opium Appetite, with Notices of Alcoholic Beverages, Cannabis Indica, Tobacco and Cocoa and Tea and Coffee, in their Hygienic Aspects and Pathological relations. By Alonzo Calkins, M. D. Philadelphia: J. B. Lippincott & Co., 1871. Buffalo: Breed, Lent & Co.

Atlantic Monthly, Advance, New York Observer, Newspaper Reporter Missionary Herald, Phrenological Journal.

The Physiological action and Therapeutic use of Chloral, by J. B. Andrews, M. D., Utica.

Bosssange's Catalogue of Anatomy, Paris, 1870.

Annual Report of the Health Officer of the City of Rochester, 1871.

The Modern Operation for Cataract. By Hasket Derby, M. D., Boston.

Amputation of Redundant Scrotum in the Treatment of Varicocele. By M. H. Henry, M. D., New York, 1871.

Seventeenth Annual Report of the New York Infirmary for Women and Children, for the year 1870. New York, 1871.

Twenty-Eighth Annual Report of the Managers of the State Lunatic Asylum for the Year 1870. Albany, 1871.

